# A comparative study for accessing primary healthcare between planning assessment and actual utilization for older adults: a case from Dalian City, China

**DOI:** 10.3389/fpubh.2023.1207098

**Published:** 2023-09-07

**Authors:** Jiayuan Bai, Wei Lu

**Affiliations:** School of Architecture and Art, Dalian University of Technology, Dalian, China

**Keywords:** healthy aging, community-oriented, primary health care, accessibility, planning assessment

## Abstract

**Introduction:**

As China has rapidly evolved into an aging society, the Chinese government has developed a community-oriented primary healthcare system to vigorously expedite the transfer of primary health care (PHC) from higher-level hospitals to community health centers (CHCs). However, current planning standards for CHCs have not considered the heterogeneity of older adults in supply-demand services, such that the areas with severe aging may comprise of underestimated levels of accessibility.

**Methods:**

This study focuses on the gap in PHC access between planning assessment and actual utilization for older adults. We conducted an empirical study in the city area of Dalian based on the check-in and survey data from CHCs during the COVID-19 pandemic. A comparison model was built to calculate matching probability using a modified Gaussian Two-Step Floating Catchment Area (G2SFCA) method.

**Results:**

As indicated by the results, the communities in the primary healthcare shortage area (PHCSA) increased 6.8% by considering the heterogeneity of older adults; these communities with underserved PHC were ignored by the current planning assessment. Based on the comparison of actual and theoretical accessibility for older adults, we found that the average matching probability was about 76.6%, which means approximately a quarter of older adults have been misestimated the accessibility of PHC.

**Discussion:**

Further analysis for the older adults with mismatched accessibility showed two causes of the gap, one is the lack of connection between the spatial distribution of facilities and the allocation of service supply, and the other is the subjective cross-catchment visit to CHCs for older adults.

## 1. Introduction

The unprecedented challenge for China's public healthcare in the 21st century is the increasingly aging population (1). According to the Seventh National Population Census (2), China has over 2.64 billion adults aged over 60 years (3), representing nearly 18.7% of the total population (4). This number is predicted to reach over 30% by 2050, which means that the population will enter an advanced stage of aging (5). The pronounced consequences in the wake of the fast-aging society involve surges in the prevalence of chronic non-communicable diseases (CNCDs) and the elevated risk of death from infectious diseases (6). As indicated by the data originating from the Global Burden of Diseases, Injuries, and Risk Factors Study (GBD) 2017, CNCDs have already been the leading cause of death in China (nearly 86.6% by population) (7). Moreover, the prevalence of CNCDs in people aged over 60 years is as high as 76%, which is much higher than people aged between 15 and 64 years (52%) (8, 9). Based on early tracking data during the COVID-19 pandemic in China, the fatality ratio (CFR) increased with age, from 0.4 % or lower in patients aged 40 years or younger but 3.6 % in patients aged over 60 years (10), and over 80% of deaths are among older adults (11). The above data fully reveal the vulnerability of older adults to health risks. In order to respond to the social structure of population aging, a powerful primary healthcare (PHC) system is regarded as the key to solving the aging problem (12–14).

**Figure 1:**
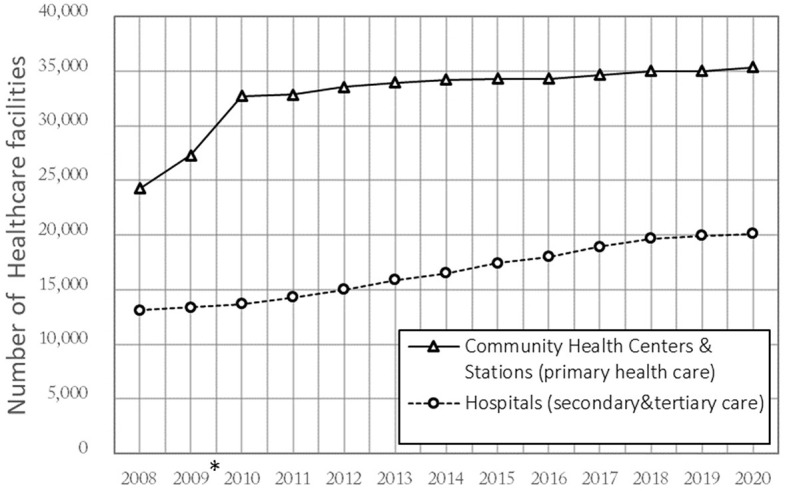
Number of healthcare facilities in China 2008–2020. *The Chinese government conducted a reform in 2009 that transferred “City-District-Street” 3-level to “City-Community” 2-level.

Back in 1978, the declaration of Alma-Ata defined PHC as the first level of contact of healthcare services (15) that which should be provided as close as possible to where people live and work (16). In most countries, PHC is primarily provided by community hospitals, clinics, and general practitioners (GPs) (17). By contrast, the PHC service in China has long been provided by high-level general hospitals (GHs) instead of community health facilities (18, 19). As societies age, China soon realized that the current hospital-centric delivery system was costly and did not serve the changing needs of the aging population, which is undergoing an epidemiological transition (20). Thus, China started a new health reform in 2009 to build a community-oriented PHC system that aims to prevent and manage chronic diseases and infectious diseases, supporting a healthy aging society. In the past decade, the government has increased funding 10-fold in community settings (20, 21); as Figure 1 shows, this promoted a transfer of PHC from GHs to community health centers and their subordinate stations (CHCs) (22). CHCs have become the core facilities of PHC, mainly providing prevention, early diagnosis, treatment, and rehabilitation (23).

**Figure 2:**
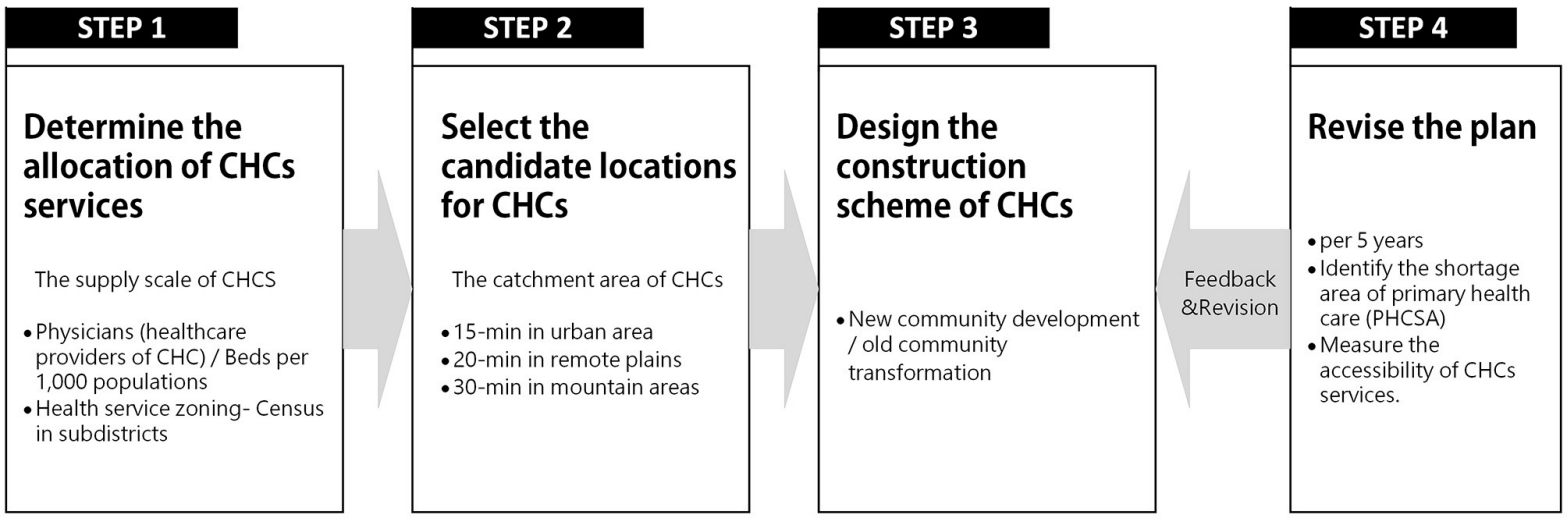
CHC planning process and standards.

The planning of CHCs, as the key part of the Urban Healthcare Facilities Plan, is established together by the regional public health department and urban planning commission, and the purpose is to facilitate the equalization of PHC and enhance the coping ability of the major epidemic and public health security events (23, 24). Figure 2 illustrates the planning process of CHCs, which includes four steps. The first step is to define the CHCs' allocation following the supply–demand equilibrium. The supply–demand scale is determined by a quantitative target of local health needs and the population size of health service zoning, including primary care physicians (PCPs) and beds per 1,000 of the population. The second step is to select the candidate locations based on the traffic and land use. The catchment area is set to a time threshold that meets the range of neighborhood living circle to ensure residents can access CHCs within a walkable distance, i.e., 15 min in urban areas, 20 min in remote plains, and 30 min in mountain areas (25, 26). The third step is to design the construction or re-construction scheme of CHCs. In general, it cooperates with the new community development or the old community transformation. In the last step, to ensure the rationality and equity of the CHC planning process, the local government will revise the plan (every 5 years) to identify the primary healthcare shortage area (PHCSA) by examining the accessibility of CHCs.

Although the current planning plays a positive role in ensuring that residents can access adequate PHC services, it has still been doubted and criticized by people because a gap exists in the results between theoretical assessment and actual utilization (14), notably for older adults (25). A limitation of the current planning is that the planning assessment using subdistricts (administrative districts) as assessment units (AUs) remains a macro-scale scheme (urban or region area scale), and there has rarely been detailed data and empirical research at the micro-scale (community scale) to validate the results. Another important limitation is that the planning standards did not consider the demographic heterogeneity in service needs. Specifically, the 2-week visiting rate in older adults was ~2.5 times the average (8). Relative to younger adults, older adults are more dependent on the community settings (27, 28) due to poorer health status and limited mobility options (e.g., walking difficulties and driving restrictions) (29). Research by Liu et al. indicated that the acceptable distance for older adults in seeking PHC was 200–600 m, and 800 m is a walking limit, which is far lower than general capabilities (30). As a logical consequence, the current planning, which defined the catchment and population sizes of CHCs based on general walkable distance and indistinctive-age population, may cause the misestimation of PHC accessibility in the areas with a high aging rate.

Based on the above inference, we searched parallel literature and found that most studies that have explored the gap in healthcare between potential access (supply) and realized access (utilization) focus on a large scale (city, county, or village scale) (31–33). In China, the restricting factor could be linked to the uncompleted constructed database at the community level and undisclosed information of CHCs (private or public–private partnership). Fortunately, closed-off management of CHCs provided a good opportunity for investigation during the COVID-19 pandemic such that our research could narrow the scale of assessment to explore the gap in PHC access between planning assessment and actual utilization for older adults. Ultimately, the aim of this study was to improve the problems of current planning and re-define the PHCSA in an aging society. Based on the data from a CHC survey in Dalian and the geographic data of PCPs, the theoretical and practical accessibility for older adults from communities with different aging levels was measured, and we identified which communities have misestimated accessibility, specifically including two aspects as follows.

(1) Whether a gap exists between the theoretical accessibility in planning assessment and the practical accessibility in real-world utilization.

(2) Whether there is a gap in accessing CHC services between the communities with different aging levels.

## 2. Methodology

### 2.1. Study area

**Figure 3:**
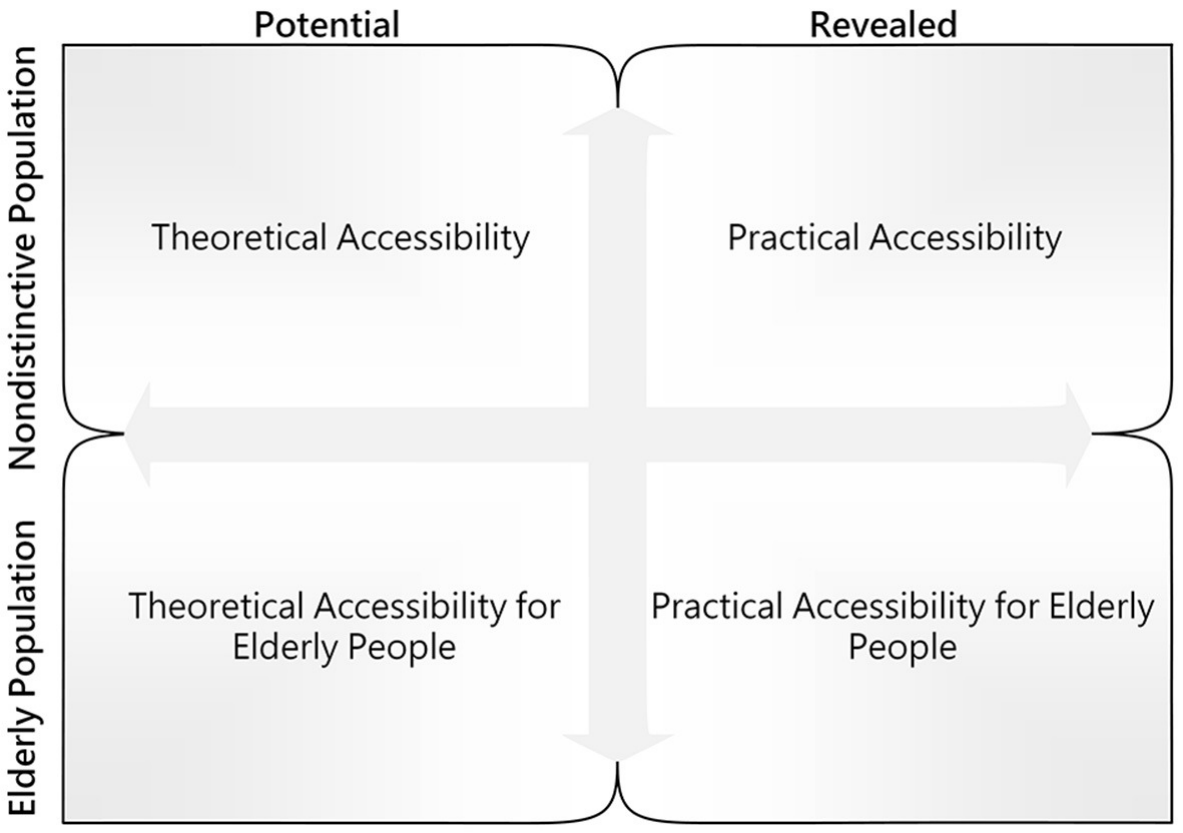
Comparison framework for accessibility.

**Figure 4:**
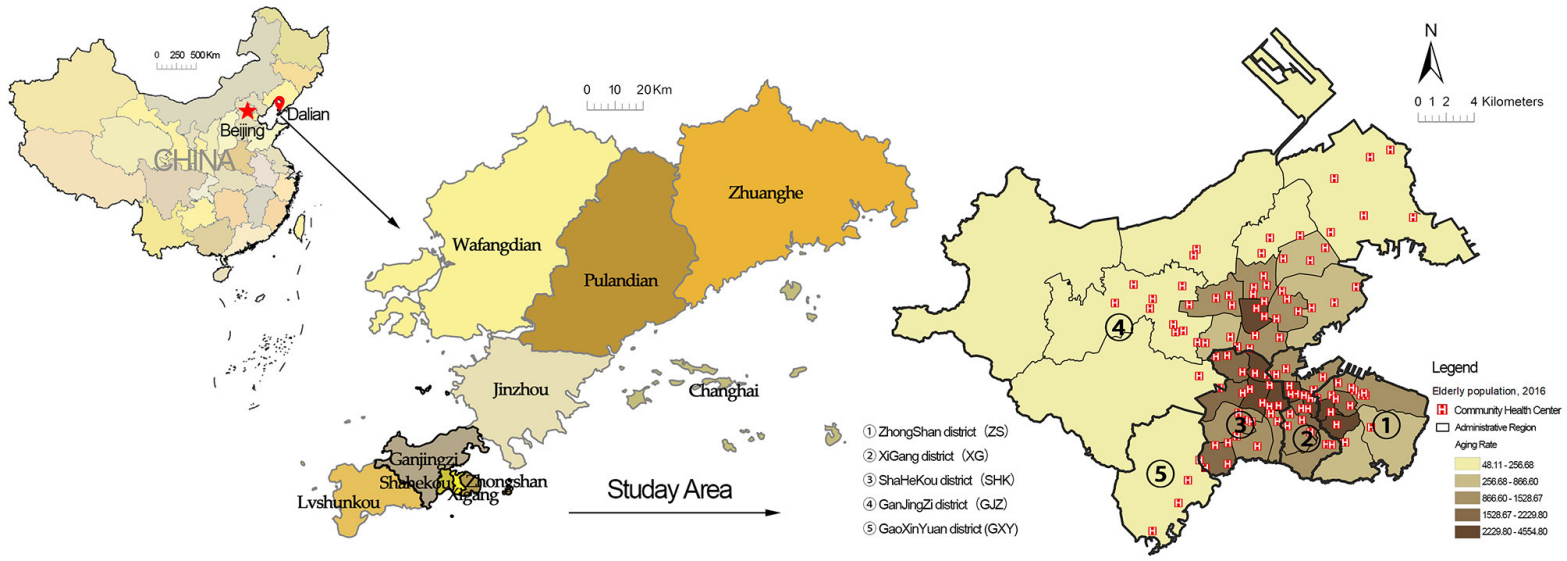
Location, aging rate, and community health center distribution of the study area. *The distribution of older adults (aged over 60 years) is based on the 2021 population census.

The empirical survey for this study was conducted in the city area of Dalian, located in the northeast peninsula of China (Figure 3). Dalian represents a unique geographic and societal setting for the research on access to PHC with the background of the aging population. Dalian has a population of more than 450,000 older adults. The aging rate of Dalian reached 24.7% in 2020 (34), which means that society has entered an advanced stage of aging. In addition, the city area of Dalian is composed of gentle slope hills and mountains such that residents mostly rely on walking for short-distance travel rather than using bicycles or electric vehicles. Thus, Dalian can serve as a typical study case to assess PHC based on a pedestrian neighborhood network, and it can also provide references for other cities in which society is at an early stage of aging. Following the planning scope of the Dalian Regional Healthcare Plan (2016–2020) (30), the study area for this study was five administrative districts (i.e., Zhongshan, Xigang, Shahekou, Ganjingzi, and Gaoxinyuan districts), including 1,359 communities and 95 CHCs (Figure 4).

### 2.2. Data and pre-processing

**Figure 5:**
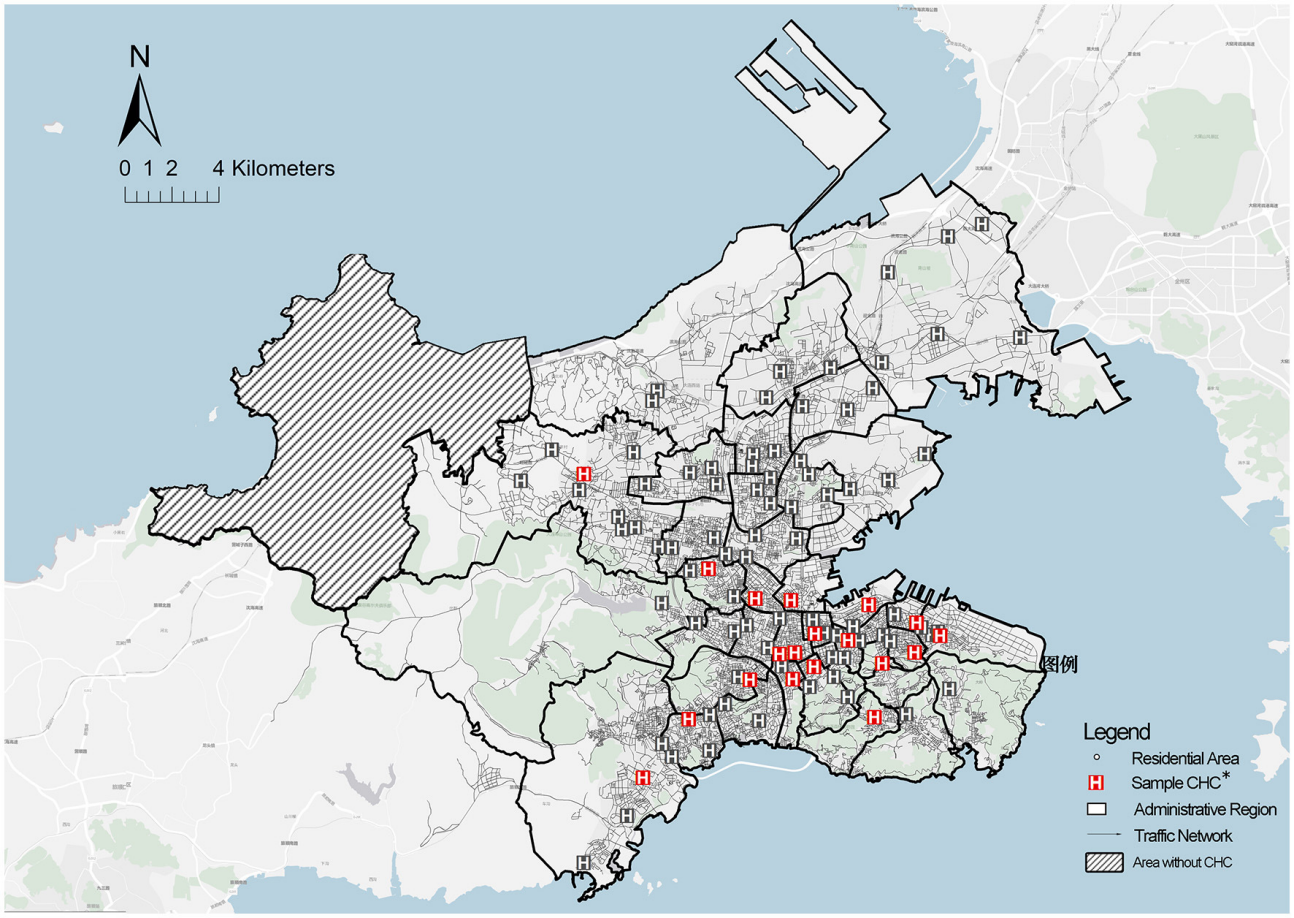
Spatial distribution of older adults at the community scale. *Community = 1,359. Data from the Seventh Population Census of Dalian.

**Figure 6:**
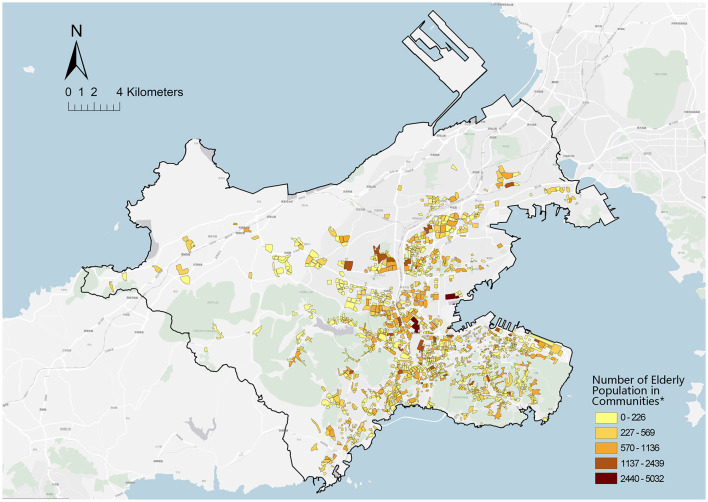
Spatial distribution of community health centers. *CHCs = 95 (including 22 CHSs); Sampled CHCs = 18; Data from Dalian Health Agency and Municipal Transportation Bureau.

The data of this study were collected from the related official information and questionnaire surveys. Data from the statistical department of the Dalian Health Commission and Dalian Planning Board were highly conducive to this study. The spatial location and basic supply of CHCs presented in Figure 5 were based on Dalian Health Statistics in 2022. Moreover, the spatial distribution of the community older adults presented in Figure 6 was obtained and then determined based on the Seventh National Population Census taken in Dalian. Furthermore, the pedestrian network adopted to measure accessibility was developed based on a revised line file provided by the Dalian Municipal Transportation Bureau. Since the original government data comprised a motor vehicle network at the city scale, the data were supplemented with more detailed internal roads in the neighborhood environment based on the satellite imagery from Baidu Maps.

The questionnaire was conducted from 18 to 20 April 2021 with the support of the administrators of public CHCs. We selected 18 CHCs that were allowed to survey based on the supply–demand ratio (Rj) for the older adults (High-Rj, Middle-Rj, and Low-Rj), including 938 older adults (aged over 60 years). During the COVID-19 epidemic period, all visitors to CHCs were asked to register their identifiable information (e.g., home address and personal identity). Accordingly, after asking respondents for permissions, we obtained their registered information and questionnaire results. All respondents were voluntary and were informed of the investigation objective. Their privacy is strictly protected.

Table 1 lists the descriptive statistics of respondents. The variables that represent the social characteristics of older adult patients are categorized into four dimensions, namely demographic characteristics, socioeconomic status, health condition, and service utilization. The first dimension reflects the aging level and composition. As Table 1 shows, the proportion of older men (46.38%) and women (53.62%) is relatively even. The old-old (aged 75–89, WHO) makes up about a fifth of the older adults, and the rest is the young-old (aged 60–74, WHO). The second dimension is the annual income of older adults, which has been proven to be an important socioeconomic constraint in health access (27). The third dimension is older adults' health conditions, including chronic diseases, long-term disease, and postoperative care, which indirectly reflects their pressing needs for PHC. For example, almost all have chronic diseases, more than half have long-term disease, and a third need postoperative care. Finally, the last dimension is the service utilization for older adults, including the visit frequency, travel time, and average access time. This directly reflects older adults' needs for CHC services; for example, 55.02% of older adults' access to CHC is beyond the preset time threshold of planning, and the old-old in poor health seldom visit CHC. To sum up, the results for the preliminary processing of survey data confirmed our suspicions that older adults have more difficulty accessing PHC in practice.

**Table 1:** Descriptive statistics of individual characteristics (*N* = 938).

	**Variable**	***N***	**%**		**Variable**	***N***	**%**
**Demographic characteristics**	**Gender**			**Health condition**	**Physical health**		
	Women	503	53.62		Very bad	29	3.09
	Men	435	46.38		Bad	181	19.3
	**Age**				Average	480	51.17
	60–65	146	15.57		Good	224	23.88
	65–70	292	31.13		Very good	24	2.56
	70–75	310	33.05		**Long-term diseases**		
	75–80	130	13.86		Not have	396	42.22
	Above 80	60	6.4		Have	542	57.78
**Socioeconomic status**	**Annual income**				**Chronic diseases**		
	< RMB¥ 10,000	43	4.58		Cardiovascular disease	316	33.69
	RMB¥ 10,000–30,000	241	25.69		Mental health issues	56	5.97
	RMB¥ 30,000–80,000	455	48.51		Osteoporosis	231	24.63
	RMB¥ 80,000–300,000	184	19.62		Hypertension	550	58.64
	Above RMB¥ 300,000	15	1.6		Diabetes	369	39.34
**Primary care of service utilization**	**Frequency**		None	2	0.21
	Almost every day	35	3.73		**Postoperative care**		
	5–10 times/month	183	19.51		No need	646	68.87
	3–5 times/month	388	41.36		Need	292	31.13
	Very seldom (>3/mo)	332	35.39				
	**Travel time**						
	< 15 min	421	44.88				
	15–20 min	285	30.38				
	20–30 min	179	19.08				
	Above 30 min	53	5.65				
	**Average access time**						
	< 10 min	53	5.65				
	< 15 min	497	52.99				
	< 20 min	883	94.14				
	< 30 min	938	100.00				

^*^Sample older adults = 938; sample CHCs = 18.

### 2.3. Measurement for the accessibility of PHC

As GIS-based spatial measurement models developed more practically (14), accessibility becomes the main basis to quantitatively assess the spatial equity and allocation rationality of public healthcare resources in urban planning, which is an aggregative index considering geographic locations and the demand–supply equilibrium distribution (35, 36). For the associated measurement methods, the series of two-step floating catchment area (2SFCA) method (37), provided by Luo et al. following the Floating Catchment Area (FCA) method, has been most extensively employed (38). The accessibility in the 2SFCA method was defined as a ratio of population to providers in the predefined health service and the population catchments (39). Furthermore, given the multi-modal traffic (40) and the diversity demand from subpopulation (39, 41), the extension models of 2SFCA (e.g., E2SFCA, KD2SFCA, 3SFCA, and i2SFCA) (36, 42–45) further improved the demand-supply scales and interaction (46) to match the real-world applications.

For high-level health services, PHC has different supply and demand sizes in accessibility measurement. First, the population size was generally smaller than 30 km, which was originally proposed by Luo et al. (47, 48) [e.g., 3 km catchment for general practitioners (GPs) in Canada (49), 4 km catchment for GPs in New Zealand (50), and population grid cell at 250 × 250 resolution in Finland (51)]. Given the population attribute, Lan et al. modeled a requirement difference among the population in ages by introducing a demand weight index (39). Second, the catchment area was calculated according to pedestrian and public transport networks instead of motor traffic networks (52). Yu et al. demonstrated the differences in healthcare accessibility measured based on the pedestrian network and motor traffic network in Shenzhen China, and they used the Delaunay triangulation skeleton model to simulate the intra-community street network that provided positive reference to our study (53). Related to the age-appropriate studies, the travel mode of older adults was considered in defining the pharmacy catchment, such as a 10-min walk or 15 min by mixed transport (54). Moreover, older adults' travel behavior was more widely considered in calculating the probability of subjective choice (39). Moreover, the reference of most significance for our study was from Di et al., where they established an indicator system to assess the spatial equity of community care services for older adults. To be specific, the accessibility of older adults was divided to three dimensions, including potential accessibility, realized accessibility, and sustainable accessibility (55).

Following the accessibility concepts proposed by Khan et al. (56) and the analytical framework for older adults accessing community care from Di et al. (55), the accessibility in this study was expressed as two dimensions and then compared horizontally and vertically. Figure 3 presents the comparison framework for accessibility. A modified Gaussian 2SFCA method was employed for accessibility measurement. Compared with other distance decay functions, the Gaussian function declined at a slower rate close to the origin, such that it was adopted to express the impedance factor for a short-distance journey. Furthermore, a comparison model was developed to determine the matching probability between theoretical accessibility and practical accessibility for older adults. The specific calculation steps are elucidated as follows:

**Step 1:** Defining the demand variance index. In this study, we set this index based on the prevalence rate of NCD patients for different ages rather than the common weight value (3–5) in existing research (57). First, the proportion of older adult patients (***D***_*k*_) is calculated by the following equation:


(1) $$\begin{array}{l} D_{k}=\frac{V_{old}\times D_{old}}{D_{all}} \end{array}$$

where ***D***_*all*_ denotes the total patients of NCD; ***D***_*old*_ is older adults, and ***V***_*old*_ is the average NCD prevalence rate of older adults aged over 60 years. The population demand ***P***_*k*_ is revised as follows:


(2) $$\begin{array}{l} P_{k}=P_{kall}+\left(DW-1\right)P_{kold}=P_{kall}+\left(\frac{D_{k}}{1-D_{k}}-1\right)P_{kold} \end{array}$$

where ***P***_*kall*_ denotes the population of location ***k***; ***P***_*kold*_ is older adults of location ***k***; and ***DW*** is the demand weight index and set to 1.94 following function (1).

**Step 2:** Computing the supply–demand ratio ***R***_***j***_, which is the ratio of service supply to all demand in the catchment area.


(3) $$R_{j}=\frac{S_{j}}{\sum_{k\in \left\{t_{kj}\leq t_{0}\right\}}P_{k}f(t_{jk})}$$

where **S**_***j***_ denotes the service supply capacity of CHC ***j (j*** **=** ***1***,…***,95)***; ***P***_*k*_ represents the total population demand of community ***k (k*** **=** ***1***,…*, n)* in the catchment area ***j***; ***t***_*kj*_ expresses the travel time between ***j*** and ***k***; ***t***_**0**_ represents the time threshold specified by the standard (23, 30, 58); and ***F(t***_***jk***_***)*** (***t***_***jk***_ ≤ ***t***_**0**_, ***t***_**0**_ **= *****30min***) is the impedance function in a walkable distance.


(4) $$f(t_{jk})=\{\begin{array}{c} 1,\;t_{ij}\leq 15\\ \frac{e^{-\frac{1}{2}\times {\left(\frac{t_{jk}}{t_{0}}\right)}^{2}-e^{-\frac{1}{2}}}}{1-e^{-\frac{1}{2}}},\;15<t_{jk}\leq t_{0}\\ 0,\;t_{jk}>t_{0} \end{array}$$

where t_ij_ represents the potential access time from community ***i*** to CHC ***j***, which is computed by the average pace of adults in general (90m/min) and older adults (58m/min) (54), and the initial impedance with no decay is set to 15 min following the planning standard (23, 25).

**Step 3:** Computing the accessibility for community ***i*** ***(i***=***1,...,1359)*** ***accessing CHC services in the catchment area*** $A_{i}^{F}$.


(5) $$A_{i}^{F}=\sum_{j\in t_{0}}R_{j}f(t_{ij})$$

where t_ij_ denotes the access time from Community ***i (i*****=*****1,...,1359)*** to CHC ***j (j***=***1,...,95)***.

**Step 4:** Using the comparison model to calculate the matching probability, which estimated the matches between potential access time and perceived travel time.


(6) $$E_{t}=\frac{\sum_{t\in \left\{t_{r}\leq t_{i}\right\}}f_{t_{r}}}{f_{j}}$$

where **E**_**t**_ denotes the matching probability between theoretical accessibility and practical accessibility; ***t***_*r*_ represents the travel time of older adults, including four time segments (i.e., 0–15, 16–20, 21–30, and above 30); ***t***_***i***_ expresses the potential access time, which is the average value of communities in the catchment area; **f**_*j*_ denotes the sampled older adults of the respective CHC, which is derived from 2% of older adults in the catchment area; **f**_*t*_*r*__ represents statistics matching ***t***_***i***_, suggesting that the time segment is not consistent with access time as the two-dimensional judgment function.


(7) $$f_{t_{r}}=\{\begin{array}{c} 1,if\;t_{r}\leq t_{i}\\ 0,if\;t_{r}>t_{i} \end{array}$$

where *f*_*t*_*r*__ denotes a binary variable; *f*_*t*_*r*__ = 1 represents two variables that are matching each other; and *f*_*t*_*r*__ = 0 if they do not.

## 3. Results

### 3.1. Comparison of two theoretical accessibility

**Figure 7:**
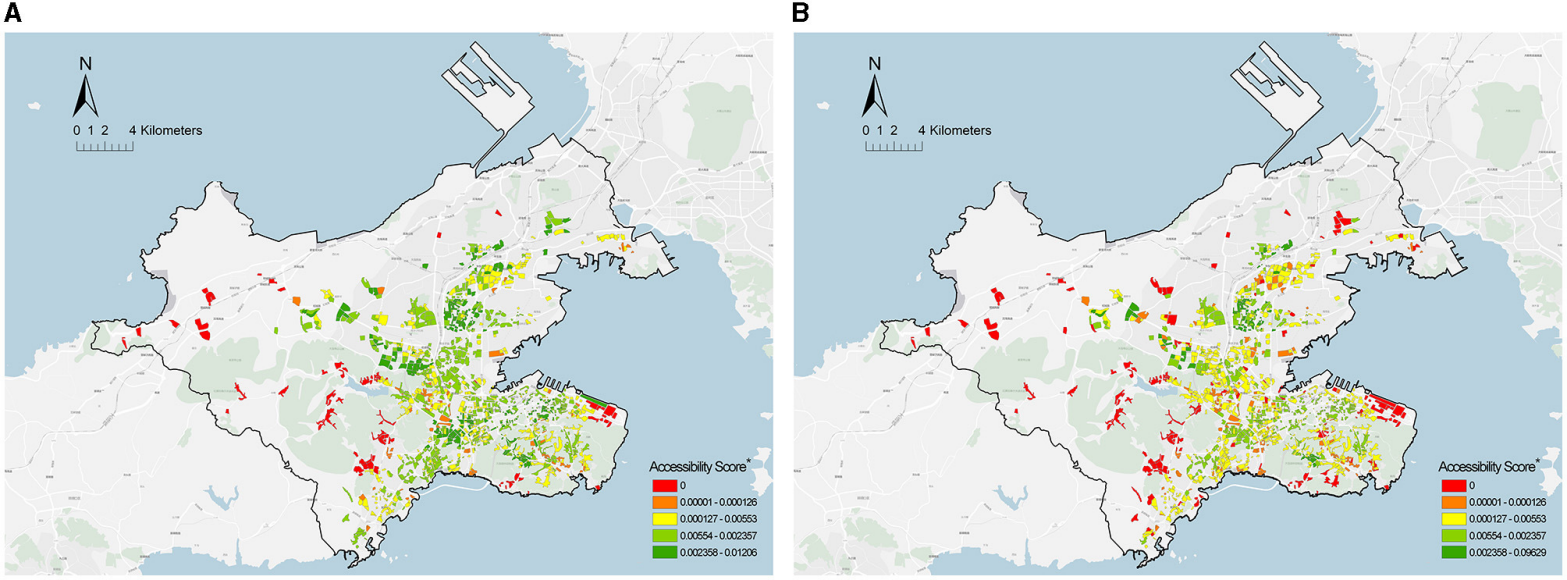
Spatial distribution of theoretical accessibility of PHC at a community scale. **(A)** All populations. **(B)** Older adult population. *Community health center = 95; Community = 1,359.

To prove that the current planning assessment using a non-distinct population age may cause a gap in accessibility measurement, the results of two theoretical accessibilities that were measured based on all populations and the older adults population were first compared, respectively. Figure 7 presents the spatial distribution of two theoretical accessibility at a community scale. The accessibility score fell into five grades (None, Low, Middle, High, and Very High) by using a natural break method (59). Furthermore, the rank of accessibility score from high to low is displayed as colors from cool to warm (red to green). The red blocks represent the communities in the PHCSA, i.e., residents' access to CHC services in the time threshold of planning standards.

The potential access time for all populations was <15 min, whereas it was nearly 23 min for the older adults population, far beyond the time threshold of the planning standard. In addition, the comparison of communities suggested that the proportion of communities with good accessibility (High level and Very High level) for the older adults population declined by 26% (61.7%−35.2%) relative to all populations, and the proportion of communities in the PHCSA increased by 6.8% (7.5%−14.3%). Notably, most communities with lower accessibility scores were distributed in the fringe areas surrounded by mountains, such that a poor pedestrian neighborhood environment was generally created (e.g., more ramps and long paths). Lastly, the degree of difference between the two theoretical accessibility was investigated. Both theoretical accessibilities displayed non-normal distributions, which were dependent on the result of the Shapiro–Wilk test. Thus, the degree of difference was obtained by paired samples of the Wilcoxon test. As depicted in Table 2, there was a significant gap between the two theoretical accessibilities (*p* < 0.001), and Cohen's d value indicated a moderate degree of difference (60).

**Table 2:** Results of the paired samples of the Wilcoxon test.

**Variable**	**SD**	**Z**	**P**	**S-W**	**Cohen's d**
Ai_pop	0.001	-	-	-	-
Ai_popold	0.001	-	-	-	-
Matches	0.001	23.052	0.000^*^	0.754 (0.000^*^)	0.763

^*^p < 0.001. Ai_pop is the accessibility score for all populations. Ai_popold is the accessibility score for the older adult population. Cohen's d indicates the effectiveness of difference; < 0.20 is a too-small effect, 0.20–0.50 is a small effect, 0.50–0.80 is a larger effect, and above 0.80 is a large effect.

### 3.2. Comparison of practical and theoretical accessibility

Based on the survey data of CHC services, we further compared the theoretical and practical accessibility for older adults. Table 3 shows the statistical results of the matching probability (E_t_) for older adults in the survey. The average matching probability (Et) was 76.6%, meaning that approximately a quarter of older adults experience a misestimated accessibility for PHC. In the association analysis between Et and the variables from the planning standards, we found six variables associated with matching probability (*p* < 0.01), namely actual travel time, potential access time, visit frequency, long-term diseases, chronic diseases, and postoperative care. The result was discussed in the following four aspects: (1) demographic characteristics (age and gender) have no associations with Et (*p* > 0.05); (2) economic status (physical health) has a positive association with Et (p < 0.05), which shows the number of matches in the lowest income group is significantly less than in other groups; (3) health conditions (chronic diseases and postoperative care) were negatively associated with Et (*p* < 0.001), and it is worth noting that the older adults who already suffer from mental health issues has the lowest Et (60%) among all chronic diseases; (4) service utilization (visit frequency, actual travel time, and potential access time) was significantly associated with Et (p < 0.001). The most interesting thing we found was that Et declined to 31% when older adults' actual travel time was beyond 20 min, whereas their potential access time showed good matching on the whole.

**Table 3:** Results of the matching probability (E_t_) in the survey.

**Variable**	**E_t_**	**X^2^**	***p***	**Variable**	**E_t_**	**X^2^**	***p***
**Gender**				**Physical health**			
Women	76.14%	0.082	0.716	Very bad	86.21%	6.918	0.140
Men	77.01%			Bad	77.35%		
**Age**				Average	75.42%		
60–65	74.66%	2.390	0.664	Good	58.33%		
65–70	77.05%			Very good	79.02%		
70–75	77.69%			**Long-term diseases**			
75–80	83.33%			Not have	78.28%	0.939	0.295
Above 80	76.55%			Have	75.28%		
**Annual income**				**Chronic diseases**			
< RMB¥ 10,000	60.47%	11.804	0.019^*^	Cardiovascular disease	76.47%	71.374	0.000^**^
RMB¥ 10,000–30,000	73.86%			Sub-optimal mental health	58.93%		
RMB¥ 30,000–80,000	77.36%			Osteoporosis	82.68%		
RMB¥ 80,000–300,000	66.67%			Hypertension	76.00%		
Above RMB¥ 300,000	82.61%			Diabetes	70.28%		
**Frequency**				None	75.36%		
Almost every day	80.00%	26.643	0.000^**^	**Postoperative care**			
5–10 times/month	68.31%			No need	80.03%	13.457	0.000^**^
3–5 times/month	72.16%			Need	68.84%		
Very Seldom (>3/mo)	85.84%						
**Travel time**							
< 15 min	100.00%	523.262	0.000^**^				
15–20 min	84.91%						
20–30 min	30.73%						
Above 30 min	0.00%						
**Average access time**							
< 10 min	50.94%	17.273	0.002^**^				
< 15 min	66.80%						
< 20 min	75.09%						
< 30 min	76.55%						

Sample size = 938. E_t_ is the matching probability. ^*^p < 0.05, ^**^p < 0.01.

Subsequently, we explored the endogenous association between the matching probability (Et) and six associative variables. Table 4 lists the results of the Pearson correlation analysis. As indicated by the result, Et was negatively correlated with the actual travel time for older adults, whereas it was positively correlated with their potential access time, suggesting that long travel times in actual utilization and the short-distance access in planning assessment can contribute to the mismatch between practical and theoretical accessibility. Moreover, we noted that the supply and demand ratio (Rj) negatively correlated with Et, but it was not correlated with the potential access time of older adults. This illustrated that there was a lack of links between the spatial distribution and service allocation of CHCs, which may also contribute to mismatching.

**Table 4:** Pearson's correlation analysis of two accessibility and matching probability.

**Accessibility**	**Correlation coefficient**	**Matching probability (Et)**	**Travel time**	**Average access time**
Practical accessibility	Age	0.019	−0.011	0.110^**^
	Annual income	0.014	0.001	0.176^**^
	Frequency	−0.152^**^	0.149^**^	0.072^**^
	Health condition	0.016	−0.031	−0.189^**^
	Postoperative care	−0.122^**^	0.166^**^	0.034
	Chronic diseases	−0.035	0.074^**^	0.102^**^
	Travel time	−0.717^**^	-	0.128^**^
Theoretical accessibility	Supply–demand ratio (Rj)	−0.351^**^	0.304^**^	0.005
	Potential access time	0.231^**^	0.128^**^	-
	Matching probability	-	−0.717^**^	0.231^**^

^*^p < 0.05, ^**^p < 0.01.

Another notable finding is that 95% of older adults with mismatched practical and theoretical accessibility selected a farther distance CHCs for PHC rather than the adjacent one. This suggests that the cross-catchment access to CHCs for older adults was probably a subjective factor of mismatched accessibility. To verify the speculation, the correlation between the proportion of cross-catchment visits (x) and the matching probability (y) was studied through a linear regression (Table 5). Linear correlation analysis indicated that they showed a significant negative correlation (*b* = −0.853, *t* = 12.617, *p* < 0.01) and passing F-test (*F* = 159.192, *p* = 0.000 < 0.05), and x can explain 90.4% variance of y.

**Table 5:** Correlation analysis of the proportion of cross-catchment visits (x) and matching probability (y).

	**Non-standardized coefficients**		**Standardized coefficients**	***t***	***P***	**VIF**	**R^2^**	**F**

	**b**	**SE**	**Beta**					
E_t_	0.882	0.038	-	23.303	0.000^**^	-	0.904	F _(1, 17)_ = 159.192, *p* = 0.000
	−0.853	0.068	−0.921	−12.617	0.000^**^	1.000		

Sample size = 938. The proportion of cross-catchment visits (x). Et is the matching probability (y). ^*^p < 0.05, ^**^p < 0.01.

**Figure 8:**
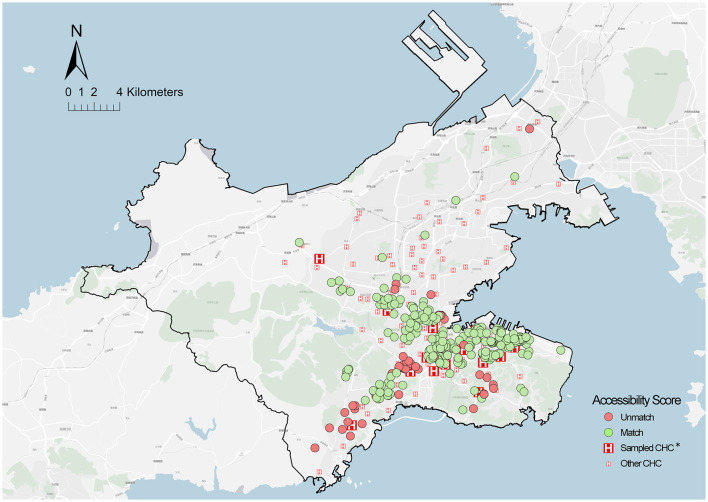
Spatial distribution of cross-catchment visit locations. *Sampled CHCS = 18; the community of sampled older adults = 579.

Figure 8 presents the spatial distribution of older adults with cross-catchment visits to CHCs, and the above-described communities are classified into two categories. The first category is communities with a high aging rate but scarce services, located on Dalian Airport Street, Dalian Square Island, China. Notably, the reason for the older adults cross-catchment to visit CHCs was to seek better medical services. The second category is communities with a high aging rate and overabundant services (primarily covering the residential areas in Chunliu Street, Malan Square, and Taoyuan Street). These areas were intensively developed in the 1990s such that the surrounding infrastructure was mostly old and creaky. Through follow-up phone calls to interview these older adults after the questionnaire, we found that they always did other activities on the way to CHCs (e.g., grocery shopping, fitness, and care massage), which led to the cross-catchment visits to CHCs.

## 4. Discussion

### 4.1. Main findings

The above results show that both comparisons have a gap in accessibility measurement for PHC, where one is between the theoretical accessibility based on all populations and the older adult population, and the other is between the practical and theoretical accessibility for older adults. The former considered the heterogeneity of older adults in the supply–demand of PHC. The results found that 10.3% of communities were identified as PHCSA, distributed across Yingchengzi, Lingshui, Xishan Reservoir, Tiger Beach, Eastport, and Dalian Bay Districts. Specifically, these areas were distributed in the fringe areas or the residential areas around mountain ranges, where poor transportation is most likely worsening an already difficult situation for older adults. The latter is based on the analysis of the variables affecting the matching probability and their endogenous effects. The analysis indicated that the factors in the aspects of socioeconomic status, health conditions, and service utilization were correlated with the matching probability of older adults. Notably, the difference of aging development between regions widened the gap between theoretical assessment and actual utilization. In addition, an unexpected finding shows that the actual travel time and potential access time have an opposite correlation with the matching probability of older adults, suggesting that both the overserved and underserved PHC affected the older adults' matches between actual utilization and planning assessment. Thus, the main reasons of mismatched accessibility can be summarized as two points. One is the lack of connection between the spatial distribution of facilities and allocation of service supply, and the other is the subjective cross-catchment visit to CHCs for older adults.

### 4.2. Policy implications

Based on the above findings, some age-friendly implications for PHC planning were proposed to improve the current gaps of accessibility. On the one hand, flexible standards should be developed for planning assessments to accommodate the regional differences in the aging level. For the allocation of CHC services, given the proportion of older adults in different health service areas, the supply amountof CHCs (physicians and beds per 1,000 people) can be increased appropriately, and some special communities should be designated (e.g., a senior-friendly community and super-aged community). Furthermore, the supply scope standard for CHCs should adjust multiple catchments by considering poor mobility among older adults, which can be determined according to the pedestrian-friendly index in the catchment area. In addition to the supply amount and scope of CHCs, the spatial aggregation of CHCs with other facilities should add to the considerations in planning standards, for instance, building a 30-min transport network between CHCs and adjacent higher-level hospitals for healthcare service at the city scale, and a collaboration network between CHCs, older adults care facilities, and other living facilities for older adult care services on a community scale. On the other hand, planning should adopt reasonable PHC zoning by using finer assessment units, specifically re-clustering the population size instead of simply using census data. It is necessary to develop a comprehensive assessment framework for PHCSA considering aging level, traffic conditions, market capacity, existing facilities, and the results of Community Health Impact Assessment (CHIA). Moreover, rather than building new facilities, ensuring the dynamics and supply–demand equilibrium of service allocation are more efficient means to improve the gap in accessibility measurement. One of the communities in the PHCSA is the community of Yingcheng District, located in a sparsely populated and high-aging fringe area, which has limited access to shopping facilities and poor transportation, where there should be an increase in dynamic services (e.g., home care, specialist visits, and treatment online). Another is the community of Eastport District, where apartments were developed with a new central business district (CBD) and are mainly used by white-collars with good mobility; therefore, PHC should combine with other public services to build multi-functional public constructions.

### 4.3. Limitations and future work

Several limitations remain in this study. Since health statistics at the community scale are non-public data in China, the samples in this study originated from older adults who were patients of CHCs, and the questionnaire did not survey older adults who were not seeking CHC services. Moreover, the actual travel time of the older adults was a self-reported subjective value, regardless of the individual differences in physical fitness and perceptive ability. Thus, more interactive tools and GPS should be adopted to measure the travel behavior of older adults in practice, and more multi-dimensional surveys should be conducted in a pedestrian environment in future research. In the aspect of analyzing factors for matching, subsequent studies should further consider environmental factors that have been confirmed to affect the accessibility for older adults (e.g., taking into account slopes and intersections).

## 5. Conclusion

Although China has been vigorously building community-oriented primary healthcare systems in recent years, spatial inequities have existed or even been ignored due to the lack of integrated, systematic, and age-appropriate planning frameworks. In the past, researchers have attempted to improve models of accessibility measures or to establish fuzzy evaluation frameworks to provide a basis for decision-making under uncertainty[62, 63]. However, few have strived to find the causes for the discrepancy between theoretical findings and practical measurements. This study demonstrated and analyzed the accessibility gap between theoretical assessment and actual utilization in PHC access for older adults and described the problem of ignoring the heterogeneity of older adults in the current planning assessment for CHCs. The proposed method has important advantages over the accessibility measures available in the literature: (i) a precise scale, (ii) combined survey experiences with planning standards, (iii) supply–demand allocation based on a population model instead of population size, and (iv) supporting sustainable planning.

## Data availability statement

The original contributions presented in the study are included in the article, further inquiries can be directed to the corresponding author.

## Author contributions

JB and WL: conceptualization, validation, and supervision. JB: methodology, software, investigation, data curation, writing—original draft preparation, visualization, and resources. WL: writing—reviewing and editing. All authors have read and agreed to the published version of the manuscript.
