# Corrigendum: A comparative study for accessing primary healthcare between planning assessment and actual utilization for older adults: a case from Dalian City, China

**DOI:** 10.3389/fpubh.2023.1359221

**Published:** 2024-01-10

**Authors:** Jiayuan Bai, Wei Lu

**Affiliations:** School of Architecture and Art, Dalian University of Technology, Dalian, China

**Keywords:** healthy aging, community-oriented, primary health care, accessibility, planning assessment

In the published article, there was an error in the correspondence. Author Jiayuan Bai was incorrectly listed as the corresponding author. The correct correspondence appears below.

Wei Lu, luweieds@dlut.edu.cn

The authors apologize for this error and state that this does not change the scientific conclusions of the article in any way. The original article has been updated.

